# GLAD-PCR Assay of R(5mC)GY Sites in the Regulatory Region of Tumor-Suppressor Genes Associated with Gastric Cancer

**DOI:** 10.32607/actanaturae.11070

**Published:** 2020

**Authors:** B. S. Malyshev, N. A. Netesova, N. A. Smetannikova, M. A. Abdurashitov, A. G. Akishev, E. V. Dubinin, A. Z. Azanov, I. V. Vihlyanov, M. K. Nikitin, A. B. Karpov, S. Kh. Degtyarev

**Affiliations:** State Research Center of Virology and Biotechnology «Vector», Novosibirsk region, Koltsovo, 630559 Russia; EpiGene LLC, Novosibirsk, 630090 Russia; Regional Clinical Oncology Center, Kemerovo, 650036 Russia; Altai Regional Oncology, CenterAltai region, Barnaul, 656049 Russia; Seversk Biophysical Research Centre, Tomsk region, Seversk, 636039 Russia

**Keywords:** gastric cancer, tumor suppressor genes, DNA methylation, GLAD-PCR assay, methyl-directed DNA endonuclease GlaI

## Abstract

At early stages of carcinogenesis, the regulatory regions of some tumor suppressor genes become aberrantly methylated at RCGY sites, which are substrates of DNA methyltransferase Dnmt3. Identification of aberrantly methylated sites in tumor DNA is considered to be the first step in the development of epigenetic PCR test systems for early diagnosis of cancer. Recently, we have developed a GLAD-PCR assay, a method for detecting the R(5mC)GY site in the genome position of interest even at significant excess of DNA molecules with a non-methylated RCGY site in this location. The aim of the present work is to use the GLAD-PCR assay to detect the aberrantly methylated R(5mC)GY sites in the regulatory regions of tumor suppressor genes (*brinp1*, *bves*, *cacna2d3*, *cdh11*, *cpeb1*, *epha7*, *fgf2*, *galr1*, *gata4*, *hopx*, *hs3st2*, *irx1*, *lrrc3b*, *pcdh10*, *rprm*, *runx3*, *sfrp2*, *sox17*, *tcf21*, *tfpi2*, *wnt5a*, *zfp82*, and *znf331*) in DNA samples obtained from gastric cancer (GC) tissues. The study of the DNA samples derived from 29 tumor and 25 normal gastric tissue samples demonstrated a high diagnostic potential of the selected RCGY sites in the regulatory regions of the *irx1*, *cacna2d3, *and *epha7 *genes; the total indices of sensitivity and specificity for GC detection being 96.6% and 100%, respectively.

## INTRODUCTION

Gastric cancer (GC) is one of the most lethal and widespread malignancies in the world. GC's ranks third among cancer mortality rates in the world; this disease is responsible for over 700,000 deaths every year [1]. According to WHO data, more than 1,000,000 new diagnoses were made and about 783,000 patients died from gastric cancer in 2018 [2].

The prognosis of the disease largely depends on its clinical stage but in general remains quite unfavorable: only 40% of patients have the potential to be cured of the disease at the time of their diagnosis. The chances of a 5-year survival period do not exceed 25–30% in most countries [3, 4], while detection of GC at early stages (IA–IB) increases that chance of survival by up to 80% or more [5, 6].

Epigenetic DNA diagnostics involving the identification of the aberrantly methylated regulatory regions of the tumor suppressor genes that are inactivated by such a modification is considered a promising tool for early cancer detection and monitoring. Such an aberrant methylation has been shown for most sporadic cancers at early stages of malignant neoplasms (more than 90% of all cases) [7, 8].

The DNA methyltransferases Dnmt3a and Dnmt3b perform *de novo *DNA methylation, including aberrant methylation. These enzymes predominantly recognize the RCGY sites (where R stands for A or G; Y stands for T or C) and modify cytosine, yielding the R(5mC) GY sequence in both DNA strands. DNA methyltransferase Dnmt1 maintains the methylation of the RCGY sites after DNA replication [9].

The methyl-directed site-specific DNA endonuclease GlaI recognizes and cleaves precisely the R(5mC) GY sites, making it a convenient tool for studying DNA methylation [10]. Based on GlaI unique specificity, we have developed a GLAD-PCR assay, a method for detecting the R(5mC)GY sites of interest even at a significant excess of DNA molecules with the corresponding non-methylated RCGY site [11].

The GLAD-PCR assay of R(5mC)GY sites displays a higher accuracy and reproducibility compared to the conventionally used method of bisulfite conversion of DNA. DNA bisulfite treatment often causes serious DNA degradation and a significant loss of material [11].

Recently, we have studied the methylation of the tumor suppressor genes in DNA samples derived from colorectal cancer tissues using the GLAD-PCR assay. Abnormal methylation of the RCGY sites in the *fbn1*, *cnrip1*, *adhfe1*, *ryr2*, *sept9I*, and *eid3 *genes was proved for more than 75% of the tumor DNA samples [12,13].

This work aimed to use the GLAD-PCR assay to detect the R(5mC)GY sites in the regulatory regions of tumor suppressor genes in DNA samples derived from gastric cancer tissues.

## EXPERIMENTAL

DNA samples intraoperatively isolated from the tumors of gastric mucosal tissue from 29 patients were used as study material. In all cases, the patients were diagnosed with gastric adenocarcinomas with varying degrees of differentiation.

Five patients had clinical stage I of the disease (T1N0-1M0, T2N0M0); 11 patients, stage II (T1N2- 3M0, T2N1-2M0, T3N0-1M0, T4aN0M0); 10 patients, stage III (T2N3M0, T3N2-3M0, T4aN1-3M0, T4bN0- 3M0); and three patients had stage IV gastric cancer (presence of distant metastases (M1) in any variants of the primary tumor size (T) and the presence or absence of metastatic lesions on regional lymph nodes (N)).

DNA samples from morphologically unchanged gastric mucosal tissue obtained from 25 GC patients during surgery on the resection line (at a distance of at least 5 cm from the macroscopically determined tumor edge) were used as the controls.

All the patients enrolled in this study have provided written informed consent.

Tissue samples obtained during surgery were placed in a test tube containing a RNA-later solution and refrigerated for 24 h at +4°C, then transferred to a freezer and stored at –20°C [12].

DNA isolation was performed using the standard phenol–chloroform method [14].

**GLAD-PCR assay**

**Fig. 1:**
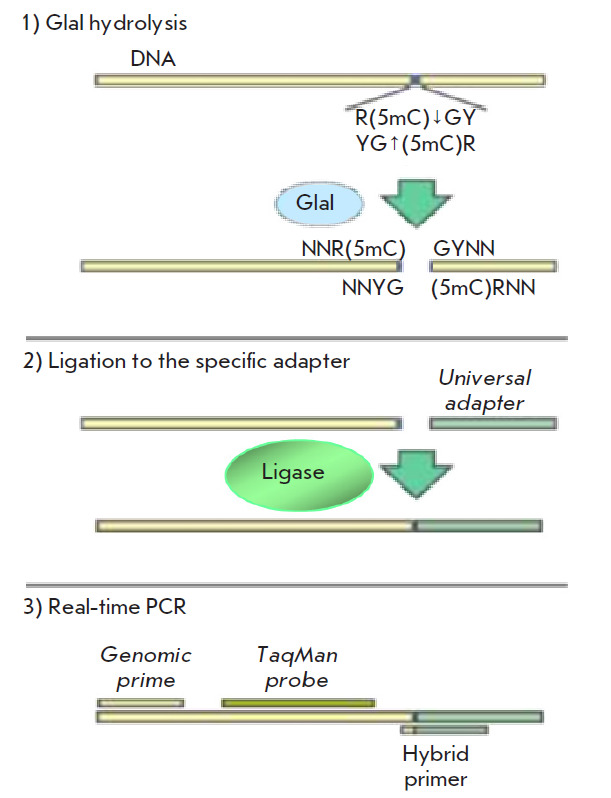
GLAD-PCR assay

The GLAD-PCR assay of DNA samples involved three stages: (1) DNA hydrolysis with the GlaI enzyme; (2) ligation of the resulting DNA hydrolysates with a universal adapter; and (3) subsequent real-time PCR using a fluorescent probe and the first primer complementary to the target DNA region, as well as the second primer corresponding to the adapter sequence and the DNA region near the determined GlaI site (*Fig. 1*).

Reagents produced by SibEnzyme Ltd were used for setting all the GLAD-PCR stages.

Enzymatic DNA hydrolysis was performed for 30 min at 30°C. The reaction mixture (volume, 21.5 μL) included 9.0 ng of the test DNA sample, 1 × SE TMN buffer (10 mM Tris-HCl (pH 7.9), 5 mM MgCl_2_, 25 mM NaCl), 2.0 % dimethyl sulfoxide (DMSO), 2.0 μg of bovine serum albumin (BSA), and 1.5 AU of GlaI.

For ligation of the hydrolysis products with the adapter (in a volume of 30.0 μL), ATP and a universal double-stranded adapter (5’-CCTGCTCTTTCATCG- 3’/3’-p-GGACGAGAAAGTAGC-p-5’) were added to each sample units to the final concentration of 0.5 μM, as well as 240 AU of highly active T4-DNA ligase. The reaction was performed for 15 min at 25°C.

At the final stage, PCR components were added to the reaction mixture to the following concentrations in the final volume 60.0 μL: 1 × SE-GLAD buffer (50 mM Tris-SO4 (pH 9.0), 30 mM KCl, 10 mM [NH_4_]_2_SO_4_), 3 mM MgCl_2_, dNTP mixture (0.2 mM each), 0.1 μg/μL BSA, the respective mixture of two primers and a probe (0.4 μM each), and 0.05 AU of SP Taq DNA polymerase. To improve the amplification efficiency of the GC-rich regions in the *tfpi2 *gene, extra DMSO was added to the PCR mixture to a total concentration of 4%.

Then, 20.0 μL of the resulting mixture was transferred into three separate microtubes and real-time PCR was performed in a CXF-96 detecting amplifier (Bio-Rad Lab., USA) according to the following program: 3 min at 95°C; 45 cycles lasting 10 s at 95°C, 15 s at 61°C (*bves*, *gata4*, *sox17*, *tcf21*) or 62°C (*cacna2d3*, *galr1*, *hs3st2*, *pcdh10*, *rprm*, *sfrp2*, *wnt5a*) or 63°C (*cdh11*, *cpeb1*, *fgf2*, *hopx*, *tfpi2*, *zfp82*), and 20 s at 72°C. To eliminate the influence of possible initial fluctuations on the shape of the amplification curve, the fluorescence of the first five PCR cycles was not recorded.

**Designing specific primers and probes**

To design the specific primers and probes, we used nucleotide sequences from the GenBank database according to the GRCh38/hg38 version of the human genome (http://ncbi.nlm.nih.gov/genbank), the Vector NTI 11.5 software family (Invitrogen, USA), and the NCBI BLAST online resource (http://blast.ncbi.nlm.nih.gov). The primer and probe structures are shown in *Table 1*.

**Table 1:** The structures of primers and fluorescent probes for a GLAD-PCR assay of gastric cancer tumor marker genes

Genea	Protein encoded namea	Chromosomal locationa	Primer/probe sequenceb
brinp1	BMP / retinoic acid inducible neural specific 1	9q33.1	FAM-CCGTAAAGTCCCCTTCGCTGGTCCC-BHQ1 GAGCCGGGATTCATGCCTGTC
bves	Blood vessel epicardial substance	6q21	CCGGCGGCATTCGTCGTT FAM-CCCTACCCGGACCGCACTTCTCGAA-BHQ1
cacna2d3	Calcium voltage-gated channel auxiliary subunit alpha2delta 3	3p21.1-p14.3	FAM-CGCACTCGGGAAAAGCACTAAGAGCCTC-BHQ1 CGAGGGAGAAGGACTGCTACCGA
cdh11	Cadherin 11	16q21	CGCTCCAGCTGGCCAGGC FAM-CTTCCCCCAACCACCATCCCGGC-BHQ1
cpeb1	Cytoplasmic polyadenylation element binding protein 1	15q25.2	CTGCCCTGGGCCTCAGTTTCC FAM-CCCCTGCGAGCGGCGGCG-BHQ1
epha7	EPH receptor A7	6q16.1	FAM-CCAAGCACGGAGCCCGGACAGTGA-BHQ1 CCCAGCCCGCGGAGGTTC
fgf2	Fibroblast growth factor 2	4q28.1	CGGGGTCCGGGAGAAGAGC FAM-CCGACCCGCTCTCTCCGCCTCATT-BHQ1
galr1	Galanin receptor 1	18q23	FAM-TGCAGCAGAGAAGCCCCTGGCACC-BHQ1 GGCGAGAGCTCTTTTGGGAGGC
gata4	GATA binding protein 4	8p23.1	CCTTTCTGGCCGGCCTCCT FAM-AGTCCCTGGACCCCAGCCCCGA-BHQ1
hopx	HOP homeobox	4q12	CGGGCAGAAGCGATGGGAGA FAM-CCCGCCGGGCTGCCCTCC-BHQ1
hs3st2	Heparan sulfate-glucosamine 3-sulfotransferase 2	16p12.2	GCCTCCCGGAGGAGTACTATGCC FAM-CACCTTCGTTTCACCGCCCCAAAGC-BHQ1
irx1	Iroquois homeobox 1	5p15.33	GCCAGGGAGCGGGTAGCGA FAM-CTCCACGGGCCTGCTTCTGCGG-BHQ1
lrrc3b	Leucine rich repeat containing 3B	3p24.1	FAM-TGCTCACCCCGTGCTGTGCAACTTG-BHQ1 GGGCTGGGGGAAGGGCAA
pcdh10	Protocadherin 10	4q28.3	CCGGCCCTTGTATCTCTGGTGC FAM-CCGCCCATCTCTGCTCCCACAACG-BHQ1
rprm	Reprimo, TP53 dependent G2 arrest mediator homolog	2q23.3	CCCCGTTCAAATTCGCAGGC FAM-CCCCCCACCCCTTCTCCCACAATGA-BHQ1
runx3	Runt related transcription factor 3	1p36.11	FAM-CCCTCCCAACTGTAGCCGGCCCC-BHQ1 CTGGGGCGATAATTCGGAATGA
sfrp2	Secreted frizzled related protein 2	4q31.3	FAM-CTCCCTTGCTCCCCCCACCCTCC-BHQ1 CCAGCCCTCCTCGGATTACCC
sox17	SRY (sex determining region Y)-Box 17	8q11.23	CGCCCTCCGACCCTCCAA FAM-TCCCGGATTCCCCAGGTGGCC-BHQ1
tcf21	Transcription factor 21	6q23.2	FAM-TGCCCCCCGACACCAAGCTCTCC-BHQ1 CCAGCCTGAGCGTGTCCAGC
tfpi2	Tissue factor pathway inhibitor 2	7q21.3	CCGAGCGGAGGGGCCTCT FAM-AGCGAGTCCCCCCTGCCAGCG-BHQ1
wnt5a	Wnt family member 5A	3p14.3	FAM-CCCTTCCCTGCCCTCCCCACAGC-BHQ1 CAGGTGTGGGGTGGGAGGGA
zfp82	Zinc finger protein 82	19q13.12	FAM-CAGCTGCAGAGAAATGGCCCTCGGTC-BHQ1 CCCCAGCATCCTCTGCCCAC
znf331	Zinc finger protein 331	19q13.42	FAM-CCGCACACTCGCTGGCCCTTTCAC-BHQ1 GCCCGATCCCGACCAGTCAC

^a^Gene symbol, protein encoded name, and chromosomal location are given in accordance with the approved guidelines from the HUGO Gene Nomenclature Committee (http://www.genenames.org);

^b^Direct genomic primer structure is indicated before the probe structure, the one for the reverse genomic primer is provided after the probe structure;

FAM – 6-carboxyfluorescein;

BHQ1 – Black Hole Quencher 1.

The hybrid primers corresponding to the R(5mC) GY sites, methylated with the highest frequency, were selected experimentally. The nucleotide sequence of each hybrid primer was 5’-CCTGCTCTTTCATCGGYNN-3’, where 15 of 5’-terminal nucleotides corresponded to the adapter, and four of 3’-terminal nucleotides (underlined) were complementary to the genomic sequence at the DNA hydrolysis site. By using hybrid primers corresponding to the terminal tetranucleotides obtained after the hydrolysis of the NNR(5mC)↓GYNN sequence, all RCGY sites – located within ~ 200 bp of the hybridization site of the fluorescent probe – in the regulatory region of each gene were analyzed. The lowest cycle threshold (Cq) value meant maximum methylation of the R(5mC)GY site [12, 13].

**Statistical analysis**

The experimental data were statistically processed using the MedCalc 15.11 software (MedCalc Software, Belgium). Based on the Cq values of the DNA samples for the analyzed RCGY sites, characteristic curves (ROC curves; Receiver Operating Characteristic Curves) were obtained with a 95% confidence interval. The area under the ROC curve (AUC) shows a correlation between the sensitivity and specificity of a diagnostic test. AUC is an integral indicator of the diagnostic efficiency of a tumor marker site. AUC is an integral indicator of the diagnostic efficiency of a tumor marker site (for the “perfect” test AUC = 1) [15].

## RESULTS

A number of candidate epigenetic GC markers have been identified thus far. Based on the results of a literature search for the epigenetically downregulated genes involved in gastric carcinogenesis, we have formed a panel of 23 tumor suppressor genes to study the methylation of the RCGY sites in their regulatory regions by GLAD-PCR assay. This list includes the *brinp1 *[16], *bves *[17], *cacna2d3 *[18], *cdh11 *[16], *cpeb1 *[19], *epha7*, *fgf2*, *galr1 *[16], *gata4 *[20], *hopx *[21], *hs3st2 *[16], *irx1 *[17], *lrrc3b *[22], *pcdh10 *[23], *rprm *[24], *runx3*, *sfrp2 *[17], *sox17 *[25], *tcf21 *[26], *tfpi2 *[27], *wnt5a *[17], *zfp82 *[18], and *znf331 *[28] genes.

**Identification of RCGY sites in DNA from GC tissues for a GLAD-PCR analysis. At this step, we used ten random DNA samples from gastric cancer tissues to select the most frequently methylated RCGY sites within the regulatory regions of tumor suppressor genes as described earlier [12, 13]**

The real-time PCR threshold cycle Cq value was used as a criterion for selecting RCGY sites promising for the GLAD-PCR assay, which should be less than 30 in at least one of the ten DNA samples from the gastric cancer tissue.

**Table 2:** RCGY sites selected for a GLAD-PCR assay, their locations, and the structure of the respective hybrid primers

Gene	Target site	Site location^a^	Hybrid primer^b^
brinp1	GCGC	chr9: 119369161–119369164	CCTGCTCTTTCATCGGCGG
bves	GCGC	chr6:105137614–105137617	CCTGCTCTTTCATCGGCGC
cacna2d3	GCGC	chr3:54120898–54120901	CCTGCTCTTTCATCGGCGA
cpeb1	GCGC	chr15: 82648343–82648347	CCTGCTCTTTCATCGGCGG
epha7	GCGC	chr6:93419955–93419958	CCTGCTCTTTCATCGGCGA
galr1	GCGC	chr18:77249828–77249831	CCTGCTCTTTCATCGGCGG
irx1	GCGC	chr5:3596424–35966427	CCTGCTCTTTCATCGGCGG
lrrc3b	GCGC	chr3:26623493–26623500	CCTGCTCTTTCATCGGCGG
pcdh10	GCGT	chr4:133152953–1331152956	CCTGCTCTTTCATCGGCGA
runx3	GCGT	chr1:24931357–24931360	CCTGCTCTTTCATCGGTGG
sfrp2	GCGC	chr4:153789030–15379033	CCTGCTCTTTCATCGGCGC
tcf21	GCGC	chr6:133889653–133889658	CCTGCTCTTTCATCGGCGA
tfpi2	GCGC	chr7:93890478–93890481	CCTGCTCTTTCATCGGCGC
znf331	GCGT	chr19:53521737–53521740	CCTGCTCTTTCATCGGTCT

^a^Site locations are given in accordance with the recent human genome assembly GRCh38/hg38;

^b^Underlined is the 3’-terminal tetranucleotide sequence (pentanucleotide one for SOX17 gene) of the hybrid primer, which is complementary to the genomic sequence at the point of GlaI hydrolysis.

According to the results of the preliminary analysis, a single RCGY site in each of the *brinp1*, *bves*, *cacna2d3*, *cdh11*, *cpeb1*, *epha7*, *fgf2*, *galr1*, *gata4*, *hopx*, *hs3st2*, *irx1*, *lrrc3b*, *pcdh10*, *rprm*, *runx3*, *sfrp2*, *sox17*, *tcf21 tfpi2*, *wnt5a*, *zfp82*, and *znf331 *genes was selected to further study the full collection of DNA samples derived from tumor (n = 29) and morphologically unchanged (n = 25) stomach mucosa tissues of GC patients (*Table 2*).

**GLAD-PCR assay of RCGY sites in DNAs from the clinical samples**

**Fig. 2:**
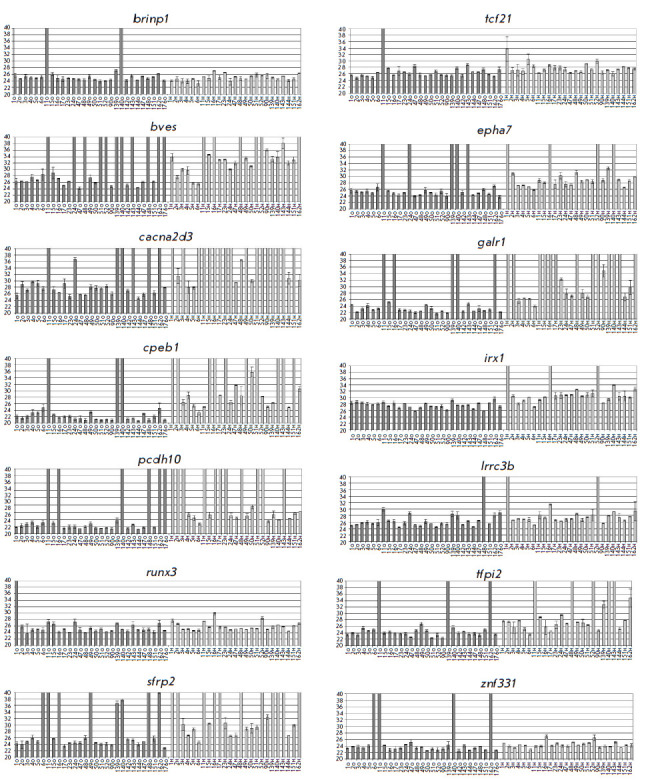
The Cq values (with the standard deviation ranges) for selected R(5mC)GY sites obtained using the GLAD-PCR assay of tissue DNAs. Sample designations are given below each diagram (T – tumor tissue, N – normal tissue)

GLAD-PCR assay of selected RCGY sites was performed in triplets of 3 ng of DNA (~ 103 copies of the studied gene region) in the reaction mixture. *Figure 2* presents the diagrams of the average Cq values for the studied RCGY sites.

The results of the analysis of the R(5mC)GY sites in the *bves*, *cacna2d3*, *cpeb1*, *epha7*, *galr*, and *tfpi2 *genes show that the Cq values (23–27) for most tumor DNA samples are on average three or more cycles lower than those for the corresponding DNA samples derived from healthy tissues. Meanwhile, for the *brinp1*, *lrrc3b, runx3, tcf21, *and *znf331 *markers, this difference in the Cq value for most DNA samples is small (less than 1.5 cycles), which makes it difficult to use them to detect tumor tissue because of a possible overlap of the range of standard deviations.

**Fig. 3:**
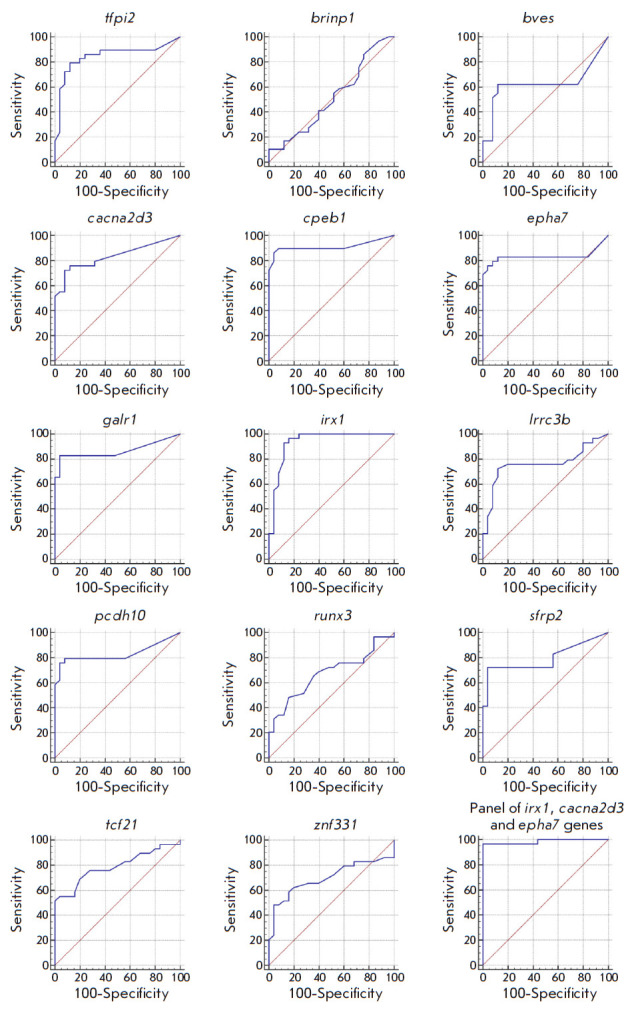
The ROC curves for a GLAD-PCR analysis of R(5mC)GY sites in GC versus normal gastric mucosa tissues

The ROC curves obtained by a statistical analysis of the experimental data from the GLAD-PCR assay for 14 RCGY sites are shown in *Fig. 3*. Meanwhile, *Table 3* summarizes the numerical results of calculated parameter values. Columns 2 and 3 indicate the number of positive results for tumor tissues for each gene and the sensitivity of determination for the site, respectively. Columns 4 and 5 show the number of negative results of the GLAD-PCR assay of the DNA samples derived from morphologically unchanged tissues and specificity in detecting tumor DNA. Column 6 lists the values of the areas under the ROC curve (AUC) expressed as a fraction of the total area of the square, indicating the standard error of measurement. Finally, column 7 lists the 95% confidence intervals for determining this parameter.

The statistical analysis of the results of the GLAD-PCR assay (*Fig. 2* and *Table 3*) shows that most of the tested markers are characterized by high sensitivity and specificity and makes it possible to differentiate between the DNA samples derived from tumor and normal tissues of gastric cancer.

The RCGY sites in the tumor suppressor genes *irx1 *and *cpeb1 *have the highest diagnostic potential; the AUC values for them are above 0.91.

The overall diagnostic characteristics of all the investigated RCGY sites were assessed using the logistic regression method (sequential inclusion/exclusion algorithm), which made it possible to select the optimal combination of markers providing the maximum area under the ROC curve and distinguish between DNA samples derived from tumor and normal tissues with the greatest efficiency. As one can see in *Table 3*, analy sis of a combination of the markers *irx1*, *cacna2d3*, and *epha7 *allows for such differentiation with 100% specificity and 96.6% sensitivity.

Thus, a diagnostic panel of RCGY sites was formed using the GLAD-PCR analysis of DNA preparations from clinical samples of tumor and normal tissues from patients with gastric cancer, which makes it possible to identify tumor tissues.

## DISCUSSION

Four molecular subtypes of GC differing in their DNA methylation profiles are known [17]. They include (a) the EBV-positive subtype, associated with the Epstein-Barr virus; (b) MSI (MLH1 silencing), characterized by functional inactivation of the *mhl*1 locus; (c) the option with stable microsatellite repeats; and (d) the subtype carrying a large number of mutations in microsatellite repeats. However, the histological type of gastric cancer is mostly adenocarcinoma (> 90%).

A significant number of genes with different biological functions have currently been identified in which the promoter regions or the first exon are methylated in gastric cancer [30]. Meanwhile, methylation of the regulatory regions of the *bves, irx1, runx3, cacna2d3, lrrc3b*, and *sfrp2 *genes presented in *Table 3* is associated with at least three subtypes of gastric cancer [17]. In the study of DNA methylation at the genome level, the bisulfite conversion method is used, followed by sequencing on the NGS platform. Sepulveda J.L. et al. applied this approach to the study of DNA preparations derived from normal mucous and tumor tissue and showed that methylation of CpG-dinucleotides is significantly increased in the *brinp1, epha7*, and *galr1 *genes in gastric cancer [16].

**Table 3:** Receiver operating characteristics for the diagnosis of GC versus normal mucosa determined by means of a GLAD-PCR assay of selected RCGY sites (sorted by AUC values)

Gene (region)	Number of detected GC samples/total number of GC samples	Sensitivity, %	Number of negative controls/total number of normal lung tissue controls	Specificity, %	AUC (standard error)	95% CI
irx1	27/29	93.1	22/25	88.0	0.934 (0.038)	0.83–0.984
cpeb1	25/29	86.2	24/25	96.0	0.911 (0.047)	0.802–0.971
galr1	24/29	82.7	24/25	96.0	0.866 (0.054)	0.745–0.943
tfpi2	23/29	79.3	22/25	88.0	0.846 (0.059)	0.721–0.929
cacna2d3	21/29	72.4	23/25	92.0	0.834 (0.054)	0.708–0.921
epha7	22/29	75.7	24/25	96.0	0.832 (0.066)	0.706–0.920
pcdh10	22/29	75.9	24/25	96.0	0.830 (0.061)	0.703–0.918
sfrp2	21/29	72.4	24/25	96.0	0.795 (0.064)	0.663–0.893
tcf21	15/29	51.7	25/25	100.0	0.790 (0.063)	0.657–0.889
lrrc3b	21/29	72.4	22/25	88.0	0.762 (0.070)	0.627–0.867
znf331	14/29	48.3	24/25	96.00	0.698 (0.075)	0.558–0.815
runx3	14/29	48.3	21/25	84.0	0.673 (0.074)	0.532–0.795
bves	18/29	62.1	22/25	88.0	0.627 (0.082)	0.485–0.755
brinp1	3/29	10.3	25/25	100.0	0.514 (0.081)	0.374–0.652
Panel of irx1, cacna2d3 and epha7 genes	28/29	96.6	25/25	100.0	0.985 (0.016)	0.907–1.000

The conclusions drawn in the listed studies were based on a comparison of the median methylation degrees in tissue samples without determining the frequency indicators of methylation or gene expression for the “normal” and “tumor” groups. Such indicators are described for the remaining five genes presented in *Table 3*.

Silencing of the *cpeb1 *gene during promoter methylation was observed in all nine studied gastric cancer cell lines and in 91% of primary tumors [19]. The same results were obtained for the *pcdh10 *gene, whose methylation was established in 82% of gastric tumors and 94% of gastric tumor cell lines [23]. Methylation of the *tfpi2 *gene promoter was also detected in more than 80% of gastric tumor samples [27]. In 71% of the pancreatic cell lines, the *znf331 *gene was turned off. This effect was also observed in a significant number of DNA samples from tumor tissues, while this gene was non-methylated in morphologically unchanged tissues, including gastric tissue [28]. Methylation of the promoter region of the *tcf21 *gene was observed only in 65% of the cases [26]. The results are presented in *Table 3* and correlate well with the previously obtained quantitative data on gene methylation in tumors of gastric cancer [19, 23, 26, 27, 28].

The list of genes with aberrant methylated sites in tumors of gastric cancer (*Table 3*) significantly differs from the earlier obtained list of genes in colorectal cancer [13]. The *sfrp2 *gene is an exception; its regulatory region’s methylation in tumor DNA is observed in both cases with almost the same frequency (72% for colorectal cancer).

**Potential of GLAD-PCR assay for gastric cancer diagnosis**

The results obtained in this study agree with the previously published data and demonstrate that a GLAD-PCR analysis allows one to determine aberrant methylated R(5mC)GY sites in the regulatory regions of tumor suppressor genes in DNA samples isolated from GC tissue. Site methylation negatively correlates with the threshold cycle value (Cq) in real-time PCR.

A combination of RCGY sites in the regulatory regions of the *irx1, cacna2d3*, and *epha7 *genes was the optimal complex marker of gastric cancer. This panel of genes allows one to achieve 100% specificity in differentiation between tumor and morphologically unchanged tissues, while the analysis sensitivity increases to 96.6% (*Table 3*).

At the moment, the so-called “liquid biopsy” technique is the most promising and actively evolving method of oncodiagnosis, which is based on an analysis of freely circulating DNA in blood. One of the main sources of such DNA in cancer patients is tumor cells, which are destroyed as a result of apoptosis and necrosis [31].

We plan to continue working with the obtained panel of markers to perform tests using DNA samples isolated from the peripheral blood of gastric cancer patients in order to develop a sensitive method for a laboratory diagnosis of gastric cancer.

## CONCLUSIONS

The R(5mC)GY sites emerging during aberrant methylation of the regulatory regions of tumor suppressor genes in DNA samples from gastric cancer tissues have been identified by GLAD-PCR assay. A panel of sites in the *irx1*, *cacna2d3*, and *epha7 *genes, which are epigenetic markers of gastric cancer, has been proposed. The high diagnostic efficiency of this panel was proved in the differentiation of DNA from morphologically unchanged and tumor tissues. The overall sensitivity and specificity of the panel are 96.6% and 100%, respectively.

We believe that the selected RCGY sites can be used to develop systems for the diagnosis of gastric cancer by the GLAD-PCR of DNA samples isolated from the blood of patients.
